# Design of Binary-Sequence Zone Plates in High Wavelength Domains

**DOI:** 10.3390/s18082604

**Published:** 2018-08-09

**Authors:** José Miguel Fuster, Sergio Pérez-López, Pilar Candelas, Constanza Rubio

**Affiliations:** 1 Departamento de Comunicaciones, Universitat Politècnica de València, 46022 Valencia, Spain; 2 Centro de Tecnologías Físicas, Universitat Politècnica de València, 46022 Valencia, Spain; serpelo1@upv.es (S.P.-L.); pcandelas@fis.upv.es (P.C.); crubiom@fis.upv.es (C.R.); 3 Departamento de Física Aplicada, Universitat Politècnica de València, 46022 Valencia, Spain

**Keywords:** zone plates, focusing, lenses, binary sequences

## Abstract

The design of zone plates is an important topic in many areas of physics, such as optics, X-rays, microwaves or ultrasonics. In this paper, a zone plate design method, which provides high flexibility in the shaping of the focusing profile, is analyzed. This flexibility is achieved through the use of binary sequences that produce zone plates with different properties and applications. It is shown that this binary-sequence method works properly at low wavelengths, but requires a modification term to work accurately in high wavelength domains. This additional term extends this powerful design method to any wavelength. Simulation results show acoustic focusing profiles for Fresnel, Fibonacci and Cantor zone plates operating at a wavelength of 1.5 mm without any distortion.

## 1. Introduction

Wave focusing is a critical application in different areas of physics, such as optics, X-rays, microwave propagation and ultrasonics [1,2,3,4]. Several devices based on both refraction and diffraction mechanisms have been proposed for implementing this application. Fresnel Zone Plates (FZPs) are particularly interesting because they are used in situations where conventional lenses are difficult to implement or planar fabrication is advantageous [5].

Traditional FZPs are capable of focusing a planar wave into a certain location with a single focus. The structure of the FZP is a set of concentric rings with increasing radius, each ring being a Fresnel region. The radius and width of each Fresnel ring depends on certain design parameters such as the desired focal length and the signal wavelength. It has been demonstrated that shifting the operating frequency modifies the focal length [6], becoming a dynamic control mechanism that increases the lens flexibility, especially in certain medical applications where fast and accurate focus shifting is critical [7].

However, conventional FZPs are severely limited in their focusing profile flexibility. Recently, new types of Zone Plates (ZPs) employing a Binary-Sequence Method (BSM) have been proposed [8,9,10,11]. Among the different lenses that can be implemented using this method, Fibonacci ZPs [8], which allow two-foci focusing profiles, and Cantor ZPs [9], which produce multiple foci reproducing the self-similarity of the ZP, should be highlighted. These new ZPs vastly increase the flexibility of the focusing profile designs and their range of applications. They have been successfully demonstrated in optics and can also be extended to other areas of physics.

It has been shown that the proposed BSM works very well for low wavelengths as is the case in optics, because it is the domain for which it was initially designed. However, it presents a significant limitation when the wavelength is increased in other physic areas such as microwaves or ultrasounds. In this work, a Modified BSM (MBSM), which uses a modification term, is proposed to be applied successfully at any wavelength, enabling the implementation of these new ZPs in any area of physics.

## 2. Zone Plate Design Methods

### 2.1. Traditional Method

When planar wave excitation is considered, the traditional Fresnel method for designing FZPs is based on Equation (1), which computes the radii of the Fresnel regions of a conventional FZP [4].

(1) $$r_{n}=\sqrt{n\lambda F+{\left(\frac{n\lambda }{2}\right)}^{2}}$$

where *F* is the FZP focal length, λ is the wavelength and n=1,...,N, *N* being the total number of Fresnel regions.

This Fresnel method can be used at any wavelength and optimizes the FZP focusing at the focal length as expected. There are two parameters that provide certain flexibility: *F* directly controls the focal length, while *N* can be used to modify the width of the focus. A higher *N* value generates a bigger FZP and results in a sharper focus, while a lower *N* value generates a smaller FZP with a wider focus. However, the design flexibility of this method is quite poor, and the applications of conventional FZPs are severely limited.

### 2.2. Binary-Sequence Method

As an alternative, Monsoriu et al. proposed a BSM based on the following coordinate transformation [8]:

(2) $$r_{n}=a\sqrt{\xi_{n}}$$

where $\xi_{n}$ is a new variable ranging from 0–1 and governed by a binary base sequence, which controls the ZP focusing profile and introduces a great degree of flexibility during the design stage. The ZP external radius, *a*, is calculated using:

(3) $$a=\sqrt{N\lambda F}$$

**Figure 1:**
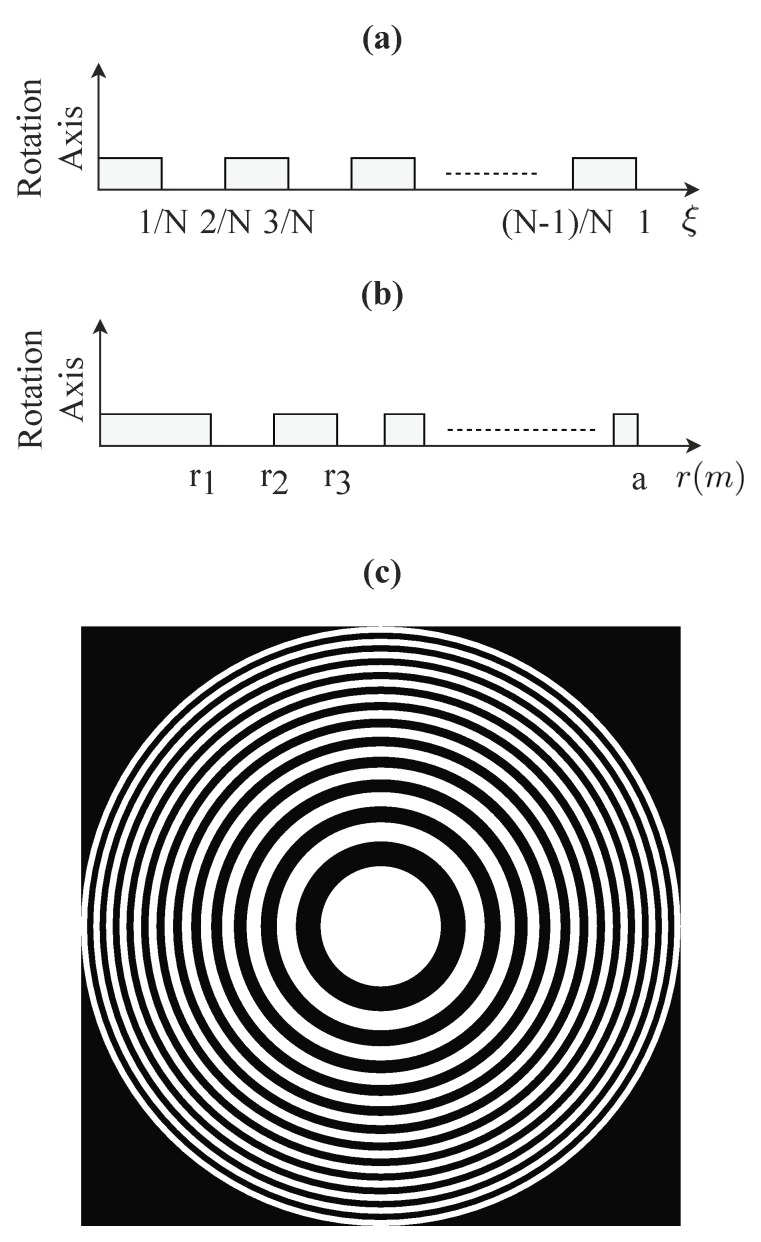
(**a**) Periodic binary sequence $\xi_{n}$. (**b**) Fresnel Zone Plate (FZP) radii $r_{n}$. (**c**) Fresnel ZP generated by rotating $r_{n}$.

Modifying the nature of the binary sequence results in a completely different focusing profile. As an example, periodic binary sequences of alternating “1” and “0” values generate conventional FZPs as shown in Figure 1. Figure 1a shows the $\xi_{n}$ variable in the base domain that corresponds to a periodic binary sequence, while Figure 1b shows the $r_{n}$ variable in the transformation domain after applying Equation (2). Figure 1c shows the conventional Fresnel ZP structure generated using the $r_{n}$ values as the radii of the Fresnel regions for N=25 and λ=633 nm.

The potential of this new BSM resides in the flexibility of the binary sequence. As shown in [8], Fibonacci binary sequences produce ZPs with two-foci focusing profiles, while Fractal binary sequences based on the Cantor set result in multi-foci focusing profiles, which can be used to minimize chromatic aberration [9]. Other binary sequences with additional properties can also be used for building ZPs with different focusing profiles [10,11]. Table 1 shows three binary sequences that result in ZPs of different natures; here, *N* states for the length of the sequence. The procedure to obtain the binary sequences for these ZPs can be found in [8,11].

**Figure 2:**
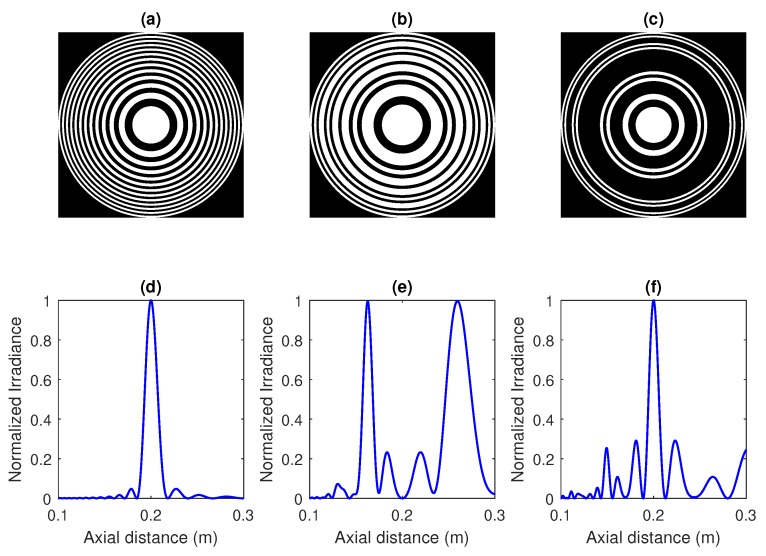
(**a**) Fresnel ZP layout and (**d**) focusing profile for N=25 and λ=633 nm. (**b**) Fibonacci ZP layout and (**e**) focusing profile for N=21 and λ=633 nm. (**c**) Cantor ZP layout and (**f**) focusing profile for N=27 and λ=633 nm.

Figure 2 shows the ZP layouts and the axial simulated focusing profiles for the previous base sequences stated in Table 1. These ZPs have been designed for a focal distance of 20 cm and a wavelength of 633 nm in optics. Focusing profiles show the normalized irradiance against the axial coordinate. Figure 2a,d correspond to the Fresnel case; Figure 2b,e correspond to the Fibonacci case; and Figure 2c,f correspond to the Cantor case. As can be observed from Figure 2d–f, the flexibility of the shape of the focusing profile has been significantly improved with this design method, and multiple foci can be achieved.

**Figure 3:**
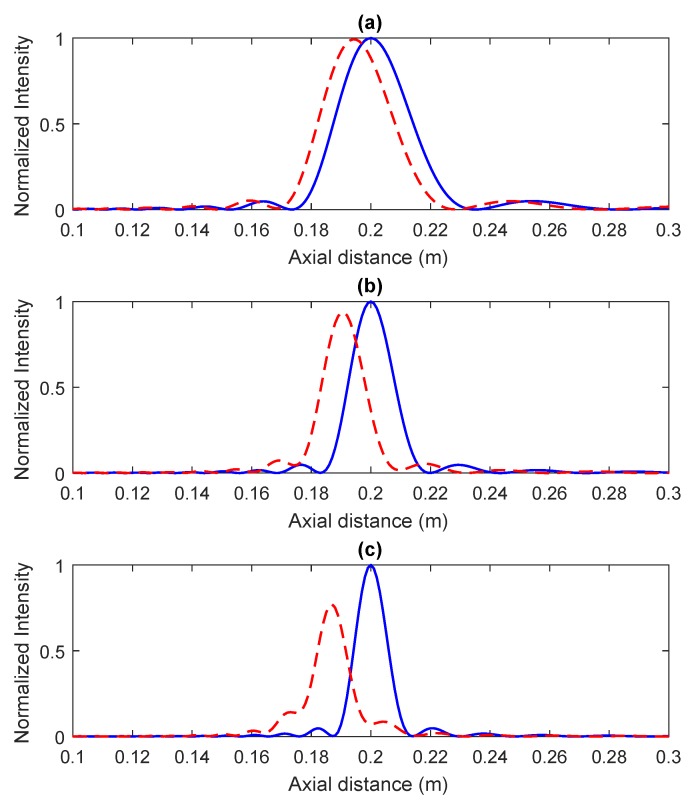
Fresnel ZP focusing profiles with N=15 (**a**), N=25 (**b**) and N=35 (**c**). Fresnel method (blue) and Binary-Sequence Method (BSM) (red). F=20 cm and λ=1.5 mm.

However, although the BSM method is very appealing, it only works properly for lower wavelengths such in the optical case. When the wavelength is increased, as in microwave or ultrasound transmission, the zone plate focusing profile becomes distorted. Figure 3 shows different focusing profiles for three FZP lenses working at a wavelength of 1.5 mm in the ultrasound domain. The blue solid lines correspond to FZPs that have been designed using the traditional Fresnel method, while the red dashed lines represent the axial focusing profiles when the BSM is employed. The normalized acoustic intensity is represented against the axial coordinate. As can be observed from Figure 3, there is a significant distortion when the BSM is employed at high wavelengths, which not only shifts the focal length, but also reduces the peak value and modifies the overall focusing profile. Figure 3a–c correspond to N=15, N=25 and N=35, respectively. As the number of Fresnel zones increases, the distortion becomes more noticeable.

In order to evaluate the limitation of the BSM in high wavelength domains, a mean error parameter, ε, is defined for the Fresnel case as:

(4) $$\varepsilon =\frac{1}{N}\sum_{n=1}^{N}\frac{\left|r_{n}^{F}-r_{n}^{BS}\right|}{r_{n}^{F}}$$

with $r_{n}^{F}$ being the Fresnel zone radii obtained from Equation (1) and $r_{n}^{BS}$ being the Fresnel zone radii computed with the coordinate transformation given by Equation (2) (BSM).

**Figure 4:**
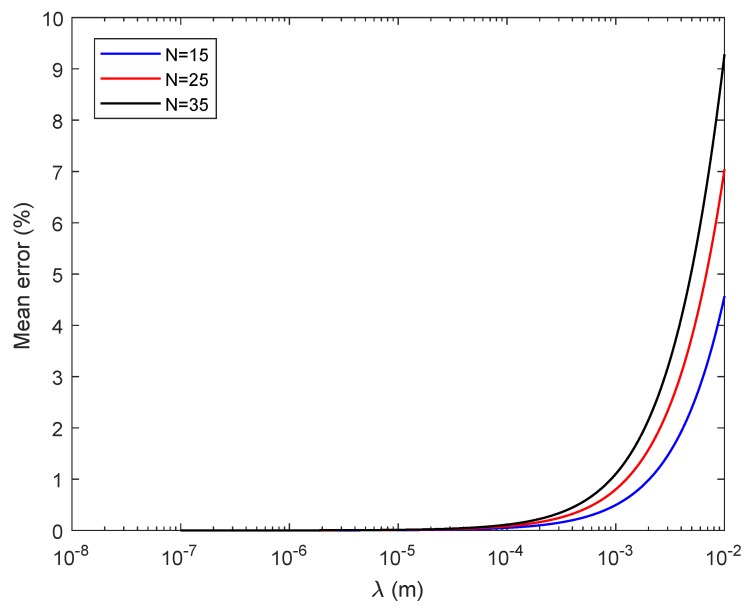
Binary-sequence FZP mean error against wavelength for N=15 (blue), N=25 (red) and N=35 (black).

Figure 4 shows the mean error against wavelength for three FZPs of different sizes. As can be deduced from this figure, the mean error increases with both wavelength and the number of Fresnel regions. When the zone plate is designed to operate in the optical domain around 633 nm, the mean error is negligible, but it becomes very significant when shifting to the ultrasound domain around 1.5 mm.

## 3. Modified Binary-Sequence Method

As can be deduced from the results shown in Figure 3, the BSM must be modified to be used in high wavelength physics. Currently, this method is applied in three steps. First, the base sequence is designed to achieve the desired focusing profile. Then, the $\xi_{n}$ sequence is obtained from the binary sequence. Finally, the coordinate transformation stated in Equation (2) is used to obtain the ZP radii.

In this work, an MBSM is proposed, which consists of adding a modification term to the values computed for the $\xi_{n}$ sequence before applying the coordinate transformation. In order to deduce this additional modification term, the Fresnel case is selected because Equation (1) allows an accurate calculation of Fresnel zone radii at any wavelength.

When the BSM is used for designing a Fresnel ZP, a periodic binary sequence must be used, which in turn generates an evenly-split $\xi_{n}$ sequence given by:

(5) $$\xi_{n}=\frac{n}{N}$$

Additionally, the $\xi_{n}$ variable can also be obtained by solving Equation (2) and is given by:

(6) $$\xi_{n}={\left(\frac{r_{n}}{a}\right)}^{2}$$

Substituting Equations (1) and (3) into Equation (6):

(7) $$\xi_{n}=\frac{n\lambda F+{\left(\frac{n\lambda }{2}\right)}^{2}}{N\lambda F}=\frac{n}{N}+\frac{n^{2}\lambda }{4FN}$$

Equation (7) gives an exact solution for the $\xi_{n}$ sequence at any wavelength in the Fresnel case, because it has been derived from Equation (1). Comparing Equations (5) and (7), the modification term can be calculated as:

(8) $$c_{n}=\frac{n^{2}\lambda }{4FN}$$

This modification term depends directly on the wavelength. Therefore, it becomes negligible for very low wavelengths as expected, but it must be considered when the wavelength is increased. When the modification term is introduced, the Fresnel zone radii obtained from the coordinate transformation Equation (2) are equal to those calculated from the reference equation in the Fresnel Case (1), and therefore, the mean error is reduced to zero. Equation (7) is as accurate as Equation (1), but much more flexible because it allows the implementation of a wide range of binary-sequence zone plates, such as Fresnel, Fibonacci and Cantor, and is not limited only to the Fresnel zone plate case as the conventional Equation (1).

**Figure 5:**
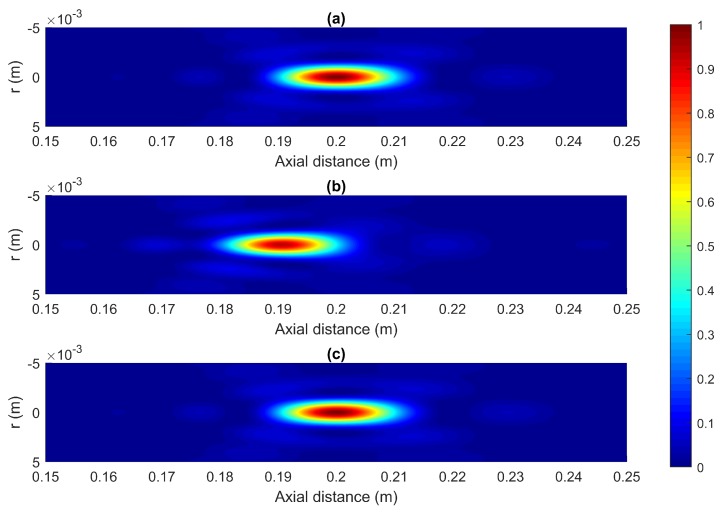
Normalized acoustic intensity maps for ZPs designed with (**a**) the Fresnel method (**b**) BSM and (**c**) Modified BSM (MBSM). N=25, F=20 cm and λ=1.5 mm.

Figure 5a–c show the normalized acoustic intensity maps for Fresnel ZPs designed using the Fresnel method, the BSM and the MBSM, respectively. The design parameters are N=25, F=20 cm and λ=1.5 mm (ultrasound domain). All values are normalized to the Fresnel method as the reference case. Figure 5b shows a focus shift in the BSM case that is amended in the MBSM case (Figure 5c).

**Figure 6:**
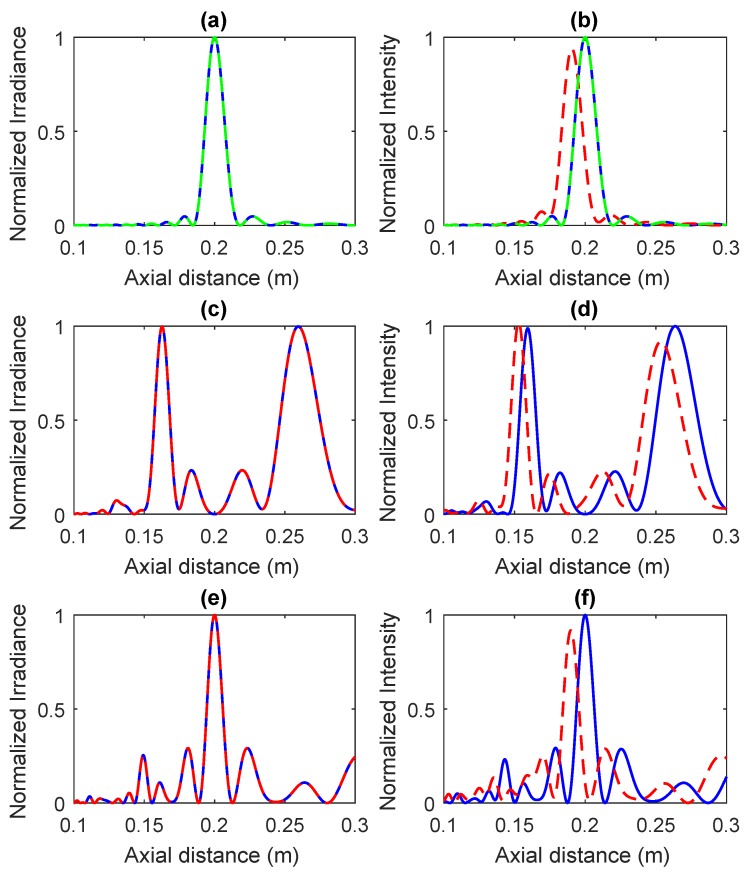
Fresnel (**a**), Fibonacci (**c**) and Cantor (**e**) ZP focusing profiles at λ=633 nm. Fresnel (**b**), Fibonacci (**d**) and Cantor (**f**) ZP focusing profiles at λ=1.5 mm. Binary-sequence method with modification term (blue), binary-sequence method without modification term (red) and Fresnel equation method (green).

Figure 6 shows the focusing profiles for three different types of ZPs at both high and low wavelengths. Figure 6a,b correspond to Fresnel ZPs with N=25; Figure 6c,d correspond to Fibonacci ZPs with N=21; and Figure 6e,f correspond to Cantor ZPs with N=27. Figure 6a,c,e are simulated at λ=633 nm, while λ=1.5 mm is used in Figure 6b,d,f. In all cases, the blue solid line corresponds to the MBSM case, while the dashed red line corresponds to the BSM case. A total of twelve different zone plates has been simulated in Figure 6, which corresponds to the combination of three parameters: the design option with three different possibilities (Fresnel, Fibonacci and Cantor), the wavelength with two cases (λ=1.5 mm and λ=633 nm) and the method employed with two options (BSM and MBSM). The number of zones is fixed for each design option: Fresnel (N=25), Fibonacci (N=21) and Cantor (N=27), but lens diameters change with wavelength or with the method used (BSM or MBSM) if working at λ=1.5 mm. Additionally, in the Fresnel case shown in Figure 6a,b, reference focusing profiles corresponding to the Fresnel method are represented as green dotted lines. These reference focusing profiles are superposed on those obtained with the MBSM, as expected, in both the optical and the ultrasound domains. As can be observed from Figure 6, base-sequence focusing profiles present no distortion in the optical domain (λ=633 nm), and the modification term is not necessary in this case, while the situation is completely different in the ultrasound domain (λ=1.5 mm), where there are significant differences between binary-sequence focusing profiles with and without modification terms. Therefore, it can be concluded that the modification term is not necessary when designing ZPs at low wavelengths, but it becomes crucial at higher wavelengths in areas such as ultrasounds or microwaves.

## 4. Conclusions

A powerful zone plate design method has been analyzed, showing its limitations when used at high wavelengths. This method employs binary sequences of different natures and allows maximum flexibility in the focusing profile by switching among periodic, Fibonacci, Cantor, Thue–Morse or m-bonacci sequences. A modification term that allows the use of this design method at any wavelength, including high-wavelength physic areas such as microwaves or ultrasounds, has been introduced. It has been shown that this modification term eliminates the existing distortion and extends this flexible zone plate design method to any wavelength.

References

## Figures and Tables

**Table 1:** Binary sequences for different ZP types.

ZP Type	N	Binary Generator Sequence
Fresnel	25	1010101010101010101010101
Fibonacci	21	101101011011010110101
Cantor	27	101000101000000000101000101
